# Correlation of renal histopathology with renal echogenicity in dogs and cats: an *ex-vivo* quantitative study

**DOI:** 10.1186/s12917-015-0415-8

**Published:** 2015-04-25

**Authors:** Alessandro Zotti, Tommaso Banzato, Maria Elena Gelain, Cinzia Centelleghe, Calogero Vaccaro, Luca Aresu

**Affiliations:** Department of Animal Medicine, Production and Health, University of Padua, Viale dell’Università 16, Legnaro (PD), 35020 Italy; Department of Comparative Biomedicine and Food Science, University of Padua, Viale dell’Università 16, Legnaro (PD), 35020 Italy

**Keywords:** Kidney, Echogenicity, Mean Gray Value, Histopathology

## Abstract

**Background:**

Increased cortical or cortical and medullary echogenicity is one of the most common signs of chronic or acute kidney disease in dogs and cats. Subjective evaluation of the echogenicity is reported to be unreliable. Patient and technical-related factors affect *in-vivo* quantitative evaluation of the echogenicity of parenchymal organs. The aim of the present study is to investigate the relationship between histopathology and *ex-vivo* renal cortical echogenicity in dogs and cats devoid of any patient and technical-related biases.

**Results:**

Kidney samples were collected from 68 dog and 32 cat cadavers donated by the owners to the Veterinary Teaching Hospital of the University of Padua and standardized ultrasonographic images of each sample were collected. The echogenicity of the renal cortex was quantitatively assessed by means of mean gray value (MGV), and then histopathological analysis was performed. Statistical analysis to evaluate the influence of histological lesions on MGV was performed. The differentiation efficiency of MGV to detect pathological changes in the kidneys was calculated for dogs and cats. Statistical analysis revealed that only glomerulosclerosis was an independent determinant of echogenicity in dogs whereas interstitial nephritis, interstitial necrosis and fibrosis were independent determinants of echogenicity in cats. The global influence of histological lesions on renal echogenicity was higher in cats (23%) than in dogs (12%).

**Conclusions:**

Different histopathological lesions influence the echogenicity of the kidneys in dogs and cats. Moreover, MGV is a poor test for distinguishing between normal and pathological kidneys in the dog with a sensitivity of 58.3% and specificity of 59.8%. Instead, it seems to perform globally better in the cat, resulting in a fair test, with a sensitivity of 80.6% and a specificity of 56%.

## Background

Abdominal ultrasound is routinely performed in animals with suspected renal disease or when increased creatinine and/or urea serum levels are detected. While disorders causing focal abnormalities such as renal cysts, nephroliths, dystrophic mineralizations, renal infarcts and solid masses are easily identified by means of B-mode ultrasound [1], architectural modifications associated with diffused parenchymal renal diseases are more difficult to assess using ultrasound. The distinction between normal kidneys and kidneys with acute or chronic damage by means of B-mode ultrasound images is currently based on subjective criteria (cortical echogenicity, shape, size and internal architecture) [1].

Cortical echogenicity is usually assessed in comparison to liver or spleen echogenicity. Since the right kidney is recessed within the renal fossa of the caudate hepatic lobe, these two structures could be compared simultaneously; similar observations may be applied for the relationship between the spleen and left kidney [1].

Increased cortical or cortical and medullary echogenicity is reported to be one of the most common signs of chronic or acute kidney disease in veterinary medicine [1]. In human medicine, a high correlation between cortical echogenicity and glomerular sclerosis, tubular atrophy, interstitial fibrosis and interstitial inflammation has been described [2].

Qualitative evaluation of the echogenicity of the renal cortex in human patients is reported to be subject to high inter- and intra-observer variation and is thus considered unreliable [3,4]. Nevertheless, the results of a comparative study between renal cortex and liver echogenicity documented a substantial agreement between qualitative and quantitative observations in healthy dogs [5].

Different factors are reported to bias the operator-based analysis of the parenchymal echogenity: 1) a multi-focal disease simultaneously affecting more than one parenchymal organ; 2) the degree of fat deposition (e.g. in the liver and kidney) that progressively increases parenchymal echogenicity; 3) the objective limited human capability to assess the actual echogenicity, as the human eye can only detect 10 to 12 shades of gray [6-8].

To overcome the above described limitations, several papers discussing the use of computer-assisted image analysis software (CAIAS) to objectively assess the echogenicity of parenchymal organs have been published in human medicine [9-19]. By contrast, to the best of the authors’ knowledge, only a few papers describing the quantitative evaluation of renal echogenicity in healthy cats and dogs are available in the literature [5-7], whereas no studies investigating the actual relationship between renal echogenicity and the degree of inflammatory or degenerative histological changes in the same pet species can be found.

It is the authors’ belief that at least 2 reasons for the scarce development of ultrasound quantitative analysis techniques in canine and feline medicine could be identified as: 1) the challenging standardization of an ultrasound-based analytical methodology in the variably-sized pet-animal patient; 2) the lack of literature regarding the correlation between CAIAS data and the status of the scanned parenchyma as determined by histopathology.

The rationale behind this funded research project (University of Padua - CPDA124900/2012) was to investigate the feasibility and usefulness of quantitative ultrasound analysis in the veterinary medical imaging field and, as part of this project, the purpose of this work was to investigate, under standardized operative conditions, the influence of different histological lesions on the echogenicity of images deriving from scans of *ex-vivo* kidneys evaluated by means of MGV.

## Methods

### Specimens

The cadavers of 68 adult dogs (30 females, 38 males; mean age 10.2 years) and 32 adult cats (9 females, 23 males; mean age 7.1 years) that died or were euthanized due to humanitarian reasons at the Veterinary Teaching Hospital, University of Padua, Italy, were the study population. Written consent was obtained from the owners. Patient history, including blood and urine analysis, was collected if available.

Each cadaver underwent a complete necropsy to determine the cause of death; all necropsies were performed within 24 hours of death. Right and left kidney tissue samples were collected from each cadaver. The kidney tissue samples consisted of half of the kidney dissected longitudinally.

### Ultrasonographic procedures

In order to standardize ultrasonographic procedures, each tissue sample was placed in an individual plastic bag containing 200 cc of tap water. The samples were scanned with a 6- to 10-MHz linear array transducer connected to a commercial sonographic scanner (Logiq P5, GE Healthcare, Milano, Italy). The following ultrasonographic settings were maintained constant throughout the scans: depth 4 cm, frequency 10 MHz, gain 50; time-gain-compensation control settings were maintained in a neutral position. The samples were imaged longitudinally (to the tissue sample) and both cortex and medulla were included in the scans.

### Image analysis procedures

Images were stored in a digital imaging and communications in medicine format (DICOM) without compression. MGV was calculated by means of an open-source CAIAS (Image J, version 1.480, National Institutes of Health, Bethesda, USA) in a manually selected polygonal region of interest (RoI). In each specimen the RoI was selected in a way to include only the renal cortex. Focal abnormalities of the renal cortex, such as mineralizations and cysts, were not included in the RoI. MGV represents the average gray value within the selected areas. In 8-bit images the gray values range from 0 to 255 (2^8^ combinations in binary code). This binary representation assumes that 0 is black and 255 is white.

### Histological analysis

The tissue samples were removed from the water-filled plastic bags and fixed in neutral-buffered 10% formalin immediately after the ultrasonographic procedures. For histopathology, renal tissue was processed routinely, and embedded in paraffin. Serial 3-mm sections were stained with hematoxylin and eosin, periodic acid-schiff, Masson’s trichrome, acid fuchsine orange-g, and periodic acid methenamine silver [20]. Congo red staining was used to confirm the presence of amyloid.

A complete histological examination was performed on each sample and lesions were classified as follows: glomerulosclerosis, interstitial nephritis, glomerulonephritis, tubular atrophy, tubular necrosis, fibrosis, glomerular lipidosis, tubular vacuolar degeneration, amyloidosis. Samples revealing no significant abnormalities were classified as “no relevant findings”. Histologically confirmed tumors were excluded from the study. A modification of the grading system used in our previous work [21] was applied and all the lesions were assessed through a semi-quantitative method on a scale ranging from 0 to 3 (where 0 = none, 1 = mild, 2 = moderate and 3 = severe).

### Statistical analysis

All the statistical analyses were performed through commercial statistical software (IBM Corp. Released 2011. IBM SPSS Statistics for Windows, Version 20.0. Armonk, NY: IBM Corp.; MedCalc for Windows, version 12.5:MedCalc Software, Ostend, Belgium). The analysis was performed separately for dogs and cats. To test the effect of histological abnormalities on MGV, grouping of samples was based on the histological findings, but due to the reduced number of some histological lesions a dichotomic variable (presence/absence) was preferred for statistical analysis. Differences between samples grouped by histological lesions and samples classified as “no relevant findings” were calculated. For normally distributed data, differences between groups were calculated using analysis of variance (ANOVA). For non-normally distributed data, the Kruskal-Wallis H test was used to investigate differences between groups. The relative contribution to echogenicity of histological parameters was evaluated through univariate analysis.

To correlate the MGV, histological lesions were included within two groups: 1) degeneration (glomerulosclerosis, tubular necrosis, fibrosis, glomerular and interstitial lipidosis, tubular atrophy, amyloidosis); and 2) inflammation (interstitial nephritis and glomerulonephritis). The semi-quantitative grades previously assessed were maintained for the statistical analysis. The Spearman rank-order correlation test was used to study the correlation between MGV and both degeneration and inflammation. For normally distributed data, differences between groups were calculated using ANOVA. For non-normally distributed data, the Kruskal-Wallis H test was used to investigate differences between groups. The Tukey-Kramer method was used for multiple comparison tests.

Lastly, the differentiation efficiency of MGV for normal (no significant findings) or pathological samples was analyzed by means of the receiver operating characteristic curve (ROC).

The variable MGV (both for dogs and cats) was tested for normality using graphic methods (histograms and Q-Q plots) and the Shapiro-Wilk test. During all statistical analyses, two-tailed P-values of less than 0.05 were considered to indicate statistically significant difference.

## Results

### Dog

Mean ± standard deviation (SD) MGVs of the dog samples classified by histological lesions are reported in Table 1. Sixty-eight kidneys (50%) were classified as “no relevant findings” and therefore were considered as normal. The most common lesion was glomerulosclerosis (19.9%), followed by interstitial nephritis (16%), fibrosis (9%), tubular atrophy (5%). Only 2 dogs affected by amyloidosis and glomerular lipidosis respectively were found and therefore such histological lesions were not considered in the statistical analysis. As expected, a mixture of histological lesions coexisted in the kidney of dogs; the presence of glomerulosclerosis was significantly correlated with the presence of fibrosis (p = 0.405; r = 0.001) tubular atrophy (p = 0.414: r = 0.001) and tubular necrosis (p = 0.346: r = 0.001). On the other hand, the presence of fibrosis was significantly related to the presence of glomerulosclerosis, interstitial nephritis (r = 0.192: p = 0.025), tubular atrophy (r = 600: p = 0.000) and tubular necrosis (r = 0.332: p = 0.001).

**Table 1:** **Descriptive statistics of the MGVs of the dog samples classified by histological lesion considered as dichotomic (presence/absence) variable**

		**MGV**		
**Histological diagnosis**	**Number of samples**	**Mean ± SD**	**Median**	**Range**
No relevant findings	68	23.44 ± 7.35	21.9	11.26-40.57
Glomerulosclerosis	32	28.53 ± 8.05	29.18	15.92-45.92
Interstitial nephritis	27	24.17 ± 6.7	23.38	11.57-38.17
Fibrosis	18	29.61 ± 7.17	29.46	18.8-44.26
Tubular atrophy	11	29.99 ± 10.6	27.5	16.69-45.92
Tubular necrosis	9	24.09 ± 8.24	21.17	11.57-36.76
Amyloidosis	2	28.18 ± 8.15	28.18	22.42-33.95

The Shapiro-Wilk normality test revealed non-normal MGV distribution in the samples; therefore, differences between groups were calculated by means of the Kruskal-Wallis H test. Statistical analysis revealed significant differences between the MGV for normal kidneys and kidneys with glomerulosclerosis (χ^2^ = 16.923; p = 0.006) and fibrosis (χ^2^ = 5.166; p = 0.025), whereas no differences were evident between normal samples and samples with interstitial nephritis, tubular necrosis, tubular atrophy. Since glomerulosclerosis and fibrosis were correlated, univariate linear regression analysis was performed to determine their relative influence on MGV. The assumptions of linearity, independence of errors, homoscedasticity, unusual points and normality of residuals were met. Only glomerulosclerosis significantly predicted MGV, *F*(2–129) = 8.247, *p* < 0.000, adj. *R*^*2*^ = 0.12. The complete results of the univariate analysis are reported in Table 2.

**Table 2:** **Results of the univariate analysis of the dog samples with MGV as dependent variable**

**Histologic diagnosis**	**B**	**Std. error**	**Beta**
Constant	22.870	0.748	
Glomerulosclerosis	4.009	1.665	0.224*
Fibrosis	4.110	2.056	0.177

B: unstandardized regression coefficient, Beta: standardized coefficient, *P value < 0.05.

**Figure 1:**
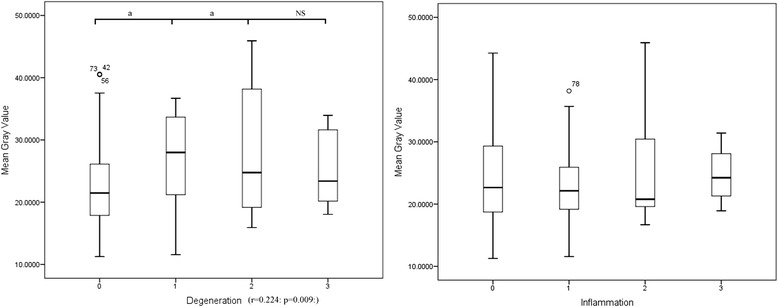
Box plot of the MGV of the canine kidney samples classified on the basis of the degeneration and inflammation histological score assigned by the pathologists (a 0 to 3 scale was used where 0 is none, 1 is mild, 2 is moderate, 3 is severe). a: significant differences (p < 0.05) between groups. NS: not significant.

Descriptive MGV statistics and box-plots (Figure 1) of the samples classified by degeneration (Table 3) and inflammation (Table 4) scores are reported. Statistical analysis revealed a significant correlation between the degeneration score and MGV (r = 0.224: p = 0.009), whereas no correlation was evident between the inflammation score and MGV (Table 3). Results of the Kruskal-Wallis H test revealed significant differences among degeneration score groups (χ^2^ = 8.289; p = 0.040) while no differences were evident among the inflammation score groups.

**Table 3:** **Descriptive statistics of the MGVs of the dog samples classified by degeneration histological score, box plot of the data are reported in Figure** 1

**Degeneration score**	**Number of samples**	**Mean** **±** **SD**	**Median**	**Range**
0	87	22.8 ± 6.86	21.47	11.26-40.57
1	22	26.78 ± 7.7	27.98	11.57-36.69
2	17	28.66 ± 10.6	24.76	15.92-45.92
3	9	24.87 ± 6.12	23.38	18.04-33.95

**Table 4:** **Descriptive statistics of the MGVs of the dog samples classified by inflammation histological score, box plot of the data are reported in Figure** 1

**Inflammation score**	**Number of samples**	**Mean** **±** **SD**	**Median**	**Range**
0	110	24.36 ± 7.7	22.65	11.57-44.26
1	14	23 ± 7.2	22.12	11.57-38.17
2	7	26.21 ± 10.47	20.76	16.69-45.92
3	4	24.7 ± 5.14	24.23	18.92-31.41

Post-hoc tests revealed significant differences between MGV of grade 0 and grades 1 (p = 0.020) and 2 (p = 0.041) degeneration scores, whereas no significant differences were evident between grades 0 and 3. Furthermore, no differences were evident between grades 1 and 2, and grades 2 and 3.

**Figure 2:**
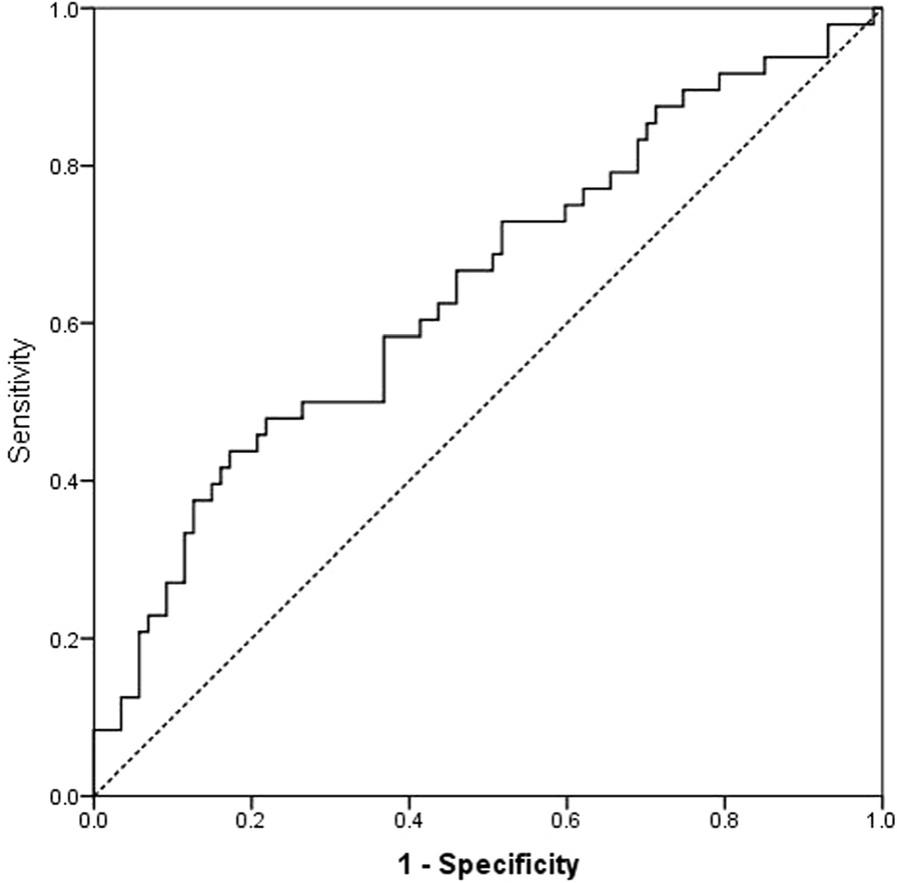
Results of the ROC curve analysis of the dog samples classified as normal or pathological (AUROC) = 0.646:p 0.005: 95% CI: 0.547-0.745). Using a cut-off value of 23.63, sensitivity was 58.3% and specificity was 59.8%.

The differentiation efficiency of MGV between normal and pathological samples, as resulted from the ROC curve analysis, is poor (area under the receiver operating characteristic curve (AUROC) = 0.646:p 0.005: 95% CI: 0.547-0.745). Using a cut-off value of 23.63, sensitivity was 58.3% and specificity was 59.8% (Figure 2).

### Cat

Mean ± standard deviation (SD) MGVs of the cat samples classified by histological lesions are reported in Table 5. Four samples were excluded because histological analysis revealed the presence of lymphoma. Differently from the dog, interstitial nephritis was the most common histological lesion, accounting for 35% of samples, followed by glomerulosclerosis (23%), tubular necrosis (8%) and fibrosis (8%). Tubular atrophy was found only in 1 sample, therefore it was not considered in the statistical analysis. Significant correlation between the histological parameters was also detected in the cat. The presence of glomerulosclerosis resulted as correlated to interstitial nephritis (r = 0.539: p = 0.000) and fibrosis (r = 0.597:r = 0.000). The presence of tubular necrosis was not correlated to any other histological lesion.

**Table 5:** **Descriptive statistics of the MGVs of the cat samples classified by histological lesion considered as dichotomic (presence/absence) variable**

		**MGV**		
**Histological diagnosis**	**Number of samples**	**Mean ± SD**	**Median**	**Range**
No relevant findings	27	18.23 ± 4.94	19.45	8.24-25.9
Interstitial nephritis	21	23.04 ± 5.4	22.72	11.12-36.68
Glomerulosclerosis	14	23 ± 5.46	22.53	12.85-36.68
Fibrosis	5	27.89 ± 5.26	26.94	22.72-36.68
Tubular necrosis	5	23.71 ± 5.38	21.62	18.34-32.38
Tubular atrophy	1	-	-	-

The Shapiro-Wilk normality test revealed normal MGV distribution in the cat samples; differences between normal samples and samples classified by histological lesion were then calculated using an ANOVA. Significant differences were evident between normal samples and no) glomerulosclerosis (F = 6.965; p = 0.012), interstitial nephritis (F = 8.886; p = 0.005), interstitial fibrosis (F = 14.919; p = 0.001) and tubular necrosis (F = 4.602; p = 0.041). Since a significant correlation between histological lesions was detected, univariate linear regression analysis was performed to determine their relative influence on MGV. The assumptions of linearity, independence of errors, homoscedasticity, unusual points and normality of residuals were met. Interstitial nephritis, fibrosis and tubular necrosis significantly predicted MGV, *F*(4, 54) = 5.040, *p* = 0.002, adj. *R*^*2*^ = 0.23. The complete results of the univariate analysis are reported in Table 6.

**Table 6:** **Results of the univariate analysis of the feline samples with MGV as dependent variable**

**Histological diagnosis**	**B**	**Std. error**	**Beta**
Intercept	18.851	0.907	
Glomerulosclerosis	−2.202	2.506	−0.174
Interstitial nephritis	4.086	1.587	0.305*
Interstitial fibrosis	4.868	2.356	0.253*
Interstitial necrosis	8.797	2.750	0.458*

B: unstandardized regression coefficient, Beta: standardized coefficient, *P value < 0.05.

**Figure 3:**
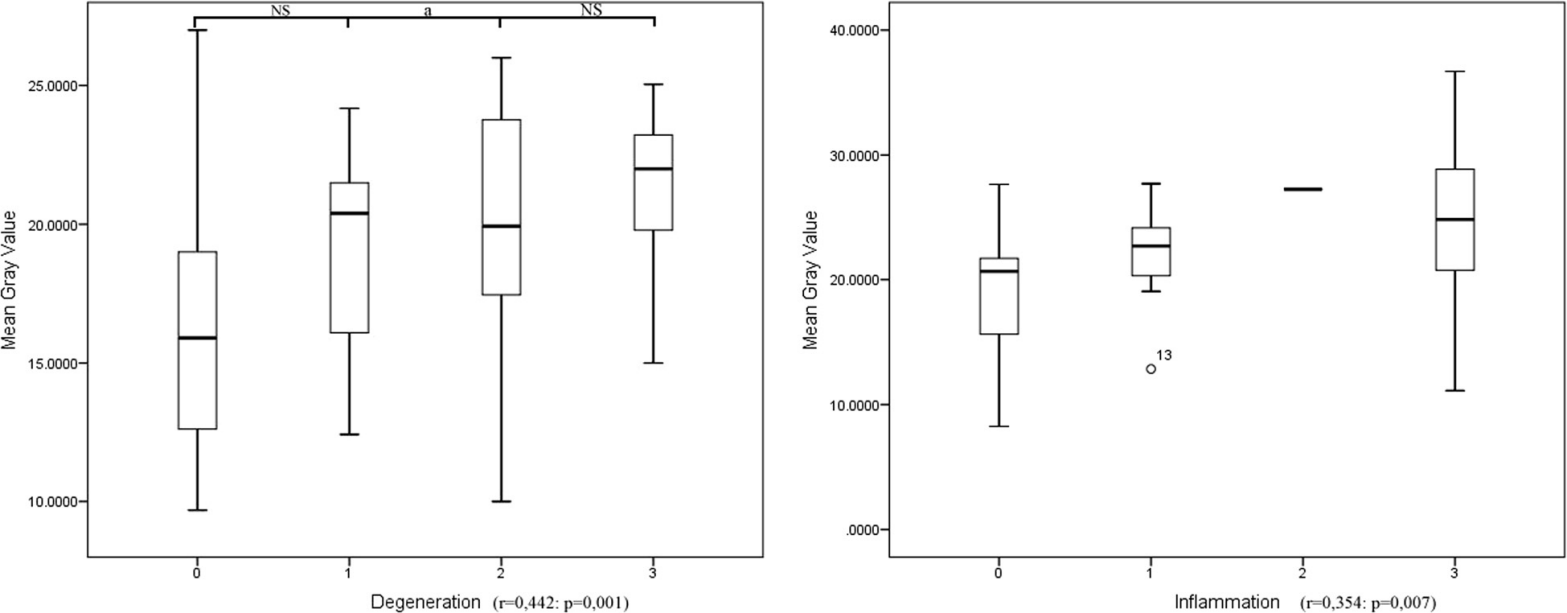
Box plot of the MGV of the feline kidney samples classified on the basis of the degeneration and inflammation histological score assigned by the pathologists (a 0 to 3 scale was used where 0 is none, 1 is mild, 2 is moderate, 3 is severe). a: significant differences (p < 0.05) between groups. NS: not significant.

Descriptive statistics and box plots (Figure 3) of the MGVs of the samples classified by degeneration (Table 7) and inflammation (Table 8) are reported. Statistical analysis revealed a significant correlation between the MGVs of the samples classified both by degeneration (r = 0.442;p = 0.001) and inflammation (r = 0.354;p = 0,007) scores. ANOVA revealed significant differences in the MGVs among groups classified both by degeneration (F = 4.727: r = 0.005) and inflammation (F = 2.846: r = 0.046) scores.

**Table 7:** **Descriptive statistics of the MGVs of the cat samples classified by degeneration histological score, box plot of the data are reported in Figure** 3

**Degeneration score**	**Number of samples**	**Mean ± SD**	**Median**	**Range**
0	36	19.33 ± 5.2	20.52	8.24-27.7
1	7	20.52 ± 4	20.88	12.85-25.48
2	11	25.24 ± 4.88	24.91	20.3-36.68
3	6	25.1 ± 5.4	25.48	18.34-32.38

**Table 8:** **Descriptive statistics of the MGVs of the cat samples classified by inflammation histological score, box plot of the data are reported in Figure** 3

**Inflammation score**	**Number of samples**	**Mean ± SD**	**Median**	**Range**
0	36	19.5 ± 5	20.66	8.24-27.65
1	13	22.17 ± 3.82	22.69	12.85-27.7
2	1	-	-	-
3	10	24.43 ± 7.6	24.83	11.12-36.68

Results of the post-hoc tests showed significant differences in MGVs only between grade 0 and grade 2 degeneration scores (Figure 3). Post-hoc tests on inflammation score were not performed since only one sample was graded as 2 on our semi-quantitative scale.

**Figure 4:**
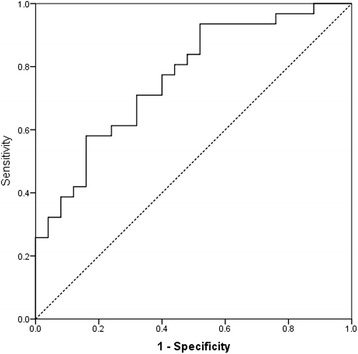
Results of the ROC curve analysis of the cat samples classified as normal or pathological (AUROC = 0.765: p 0.001: 95% CI: 0.642-0.889). Using a cut-off value of 20.16 the sensitivity was 80.6% and specificity was 56%.

MGV differentiation efficiency between normal and pathological samples, as resulted from the ROC curve analysis, is fair (AUROC = 0.765:p 0.001: 95% CI: 0.642-0.889). Using a cut-off value of 20.16 the sensitivity was 80.6% and specificity was 56% (Figure 4).

## Discussion

Technical and patient-related factors potentially biasing quantitative ultrasonography are mainly linked to RoI depth (the distance between the tissue and the probe), to the amount and type of tissue overlying the RoI (i.e. the amount of subcutaneous or visceral fat), and to the ultrasonographic settings (especially to Gain) [6].

Previous studies aimed at an objective evaluation of renal echogenicity in dogs and cats were performed exclusively on healthy animals and the echogenicity was evaluated in a comparative manner (to a phantom [6] or to the liver [5]).

To obtain reliable data without the need for a phantom and/or comparison to other parenchymal organs and, at the same time, to avoid patient and technical related biases, we analyzed *ex-vivo* kidneys under standardized experimental conditions. Furthermore, we correlated the MGVs, as measured through CAIAS, not only to normal but also to pathological kidneys, as diagnosed by histopathology. To the best of the authors’ knowledge, this is the first report in veterinary medicine correlating sonographic findings with renal histopathology and thus with the severity of degenerative and inflammatory changes.

In dogs only glomerulosclerosis was an independent determinant of echogenicity. In cats, all the considered parameters were correlated with echogenicity but only interstitial nephritis, fibrosis and tubular necrosis were independent determinants of echogenicity. Moreover, histological lesions accounted for 23% of total variability in the feline samples, while histological lesions explained only 12% of variability in dogs. The remaining 77% of variability in cats and 88% in dogs could be related to an individual variation effect or to the type of image analysis performed. An *in-vivo* study in human medicine quantitatively assessing the influence of pathological changes (glomerulosclerosis, fibrosis, tubular atrophy and interstitial inflammation) on the echogenicity of the kidney reported that only 20% of the total variability was explained by the histological parameters [2].

Individual differences in the amount of fat vacuoles in the renal tubular epithelium are described to substantially affect the echogenicity of kidneys in healthy cats [22]. Since the different amount of fat vacuoles is considered a para-physiological variation, it was not reported within histological results. It is very likely, however, that such individual variations might account for part of the variability in the univariate analysis unexplained by the histological parameters. On the contrary, there is not, to the best of our knowledge, enough information reported in the literature to justify the low influence of histological lesions on canine MGVs.

Statistical analysis of the degenerative and inflammatory changes evaluated in our semi-quantitative histological score system also revealed different results in dogs and cats. In dogs, kidney echogenicity rapidly increases with mild degenerative changes but decreases and becomes similar to that of normal kidneys in the presence of more severe lesions (Figure 1). Furthermore, inflammatory changes did not yield any significant variation in renal echogenicity. The MGVs for feline kidneys have a more linear correlation with the degeneration and inflammation histological scores than the canine samples.

It is reported that the clinical distinction between normal and chronically degenerated kidneys solely on the basis of echogenicity may be difficult and that other ultrasonographic characteristics (such as shape, size, contour and internal architecture) might be helpful [1]. In fact, as a result of the ROC curve analysis of the dog samples, MGV is a poor test to distinguishing between normal and pathological kidneys with a high sensitivity but low specificity. In the cat, MGV seems to perform globally better, resulting as a fair test, despite the very high specificity and low sensitivity.

It is the authors’ opinion that the differences observed between dogs and cats may be related to different factors: 1) the relative low number of samples demonstrating the presence of one or more histological lesion (e.g.: fibrosis and tubular necrosis in the cat, tubular atrophy and tubular necrosis in the dog); 2) the different number of samples between dogs and cats; 3) the reported differences in the pathogenesis of chronic kidney disease in dogs and cats [23], which may influence echogenicity differently. With reference to this latter point, no agreement has been reached to date regarding the variations in the pathogenesis of chronic kidney disease in dogs and cats, and different studies report different histological lesions to be correlated with disease severity [23,24].

The present study was performed on *ex-vivo* samples and factors potentially biasing the analysis have been avoided; further *in-vivo* studies, possibly involving a larger number of subjects, are required to test whether the MGV analysis could be shifted to the clinical field.

## Conclusions

This is the first report in veterinary medicine quantitatively assessing through a standardized procedure the influence of renal histopathology on the echogenicity of the kidneys. Mean gray value or renal cortical echogenicity is mainly influenced by glomerulosclerosis and fibrosis in dogs and by interstitial nephritis, tubular necrosis and fibrosis in cats. The global influence of histological lesions on renal echogenicity was higher in cats than in dogs. The MGV of the canine kidneys rapidly increases in relation to mild degenerative lesions but decreases as the severity of the lesions progresses. Inflammatory lesions show no influence on echogenicity. The echogenicity of the feline kidneys increases linearly both with inflammatory and degenerative changes.

## Abbreviations

ANOVA: Analysis of variance
AUROC: Area under the receiver operating characteristic curve
CAIAS: Computer-assisted image analysis software
DICOM: Digital imaging and communications in medicine
MGV: Mean gray value
ROC: Receiver operating characteristic curve
RoI: Region of interest
